# Philadelphia Almshouse Infirmary

**Published:** 1829-08

**Authors:** 


### PHILADELPHIA ALMSHOUSE INFIRMARY.

*Gangrene of the Right Lung.* By Dr. Samuel Jackson.

Richard Harrison, aged forty-three years, was admitted on the 20th of November, in the cells, with mania a potu, for which he was treated with the usual remedies, and entirely recovered. On the 26th, complained of pain in his right side; pulse frequent and tense: was ordered to be freely cupped over the seat of pain.

Next morning felt greatly relieved; but, his eye being much inflamed, and having an ulcer on the cornea, he was ordered to the Eye ward, where he was principally treated for that affection. The cups were repeated twice to his chest with advantage; he also made use of a common cough mixture, and drank freely of flaxseed tea, containing Supertart. of Potash ʒij., Tart. Antim. gr. ij.; his diet being gum-water and gruel. Was removed into Clinical ward.

December 12th.—On examination of his chest, percussion elicits a very flat sound all over the right side, and no respiration can be heard in that lung; he coughs very often, and brings up with difficulty a viscid, thick expectoration, some of which adheres to the vessel when reversed; tongue covered with a thick, dark fur; pulse frequent and irritated; bowels much disordered; abdomen tense; breath fetid.—Ordered Tinct. Opii ʒiv.; Tart. Acid. gr. xij.; Water ℥ij., teaspoonful occasionally.

Evening.—Much worse; cough almost constant. On examination, gangrene of the right lung suspected.—Ordered opiates to be freely given.

13th.—Continues to sink: respiration can be heard all over the room; pulse very small and frequent; tongue very foul, and breath extremely fetid; diarrhœa has supervened. He continued to take opiates; but he gradually sunk, and died about twelve P.M.

Case reported by Dr. CLARK.

*Autopsy.*—An examination was made of the body, but limited merely to the thorax, in order to determine the condition of the lungs. The left collapsed when the sternum was raised, and was perfectly natural in structure. The right exhibited the appearance of a soft grumous mass, of a black colour. No adhesions of the pleura existed. On attempting to remove the lung from the cavity, it was ruptured, and the lower and middle lobes exhibited the semblance of a quantity of putrid coagulated blood, and exhaled a most offensive odour.

*Remarks.*—This case offers another instance of the extreme disorganization which may occur in the viscera of drunkards, without exhibiting premonitory symptoms to induce a suspicion of the intensity of the disease. Harrison was brought to the infirmary, for the third or fourth time, with mania a potu. At this period he had no cough of consequence, and attention was not directed to his thoracic organs. It is, notwithstanding, most probable that he was then labouring under the pulmonary affection that terminated his existence. As the symptoms of mania subsided, some cough was noticed, but it could not have been urgent, as the resident physician did not direct my notice to it. Being transferred to the Eye ward, he was not under the observation of the medical attendants until he was brought into the Clinical wards, two days previous to his death.

The affection of the right lung approached, in its anatomical characters, to the uncircumscribed gangrene of Laennec, which he states "may be placed in the number of the most rare of the organic diseases." This morbid organic affection occurred but twice in the extensive researches of this eminent cultivator of pathological anatomy in a space of twenty-four years; and but five or six instances of it had been met with in the hospitals of Paris.

At first it might be looked upon as pulmonary apoplexy; but this disease is always in circumscribed spaces of the lung, and is accompanied with profuse hæmoptoë, which in this case was absent.—*American Journal of Med. Sciences.*

